# Bone Marrow-Derived Vasculogenic Mesenchymal Stem Cells Enhance In Vitro Angiogenic Sprouting of Human Umbilical Vein Endothelial Cells

**DOI:** 10.3390/ijms24010413

**Published:** 2022-12-27

**Authors:** Hyun Hee Jang, Youngsook Son, Gabee Park, Ki-Sook Park

**Affiliations:** 1 Graduate School of Biotechnology, Kyung Hee University, Yongin 17104, Republic of Korea; 2 Department of Biomedical Science and Technology, Graduate School, Kyung Hee University, Seoul 02447, Republic of Korea; 3 East-West Medical Research Institute, Kyung Hee University, Seoul 02447, Republic of Korea

**Keywords:** bone marrow, vasculogenic mesenchymal stem cells, HGF, bFGF, angiogenesis

## Abstract

Vasculogenic properties of bone marrow-derived mesenchymal stem cells (MSCs) have been reported, but it is still unclear whether the vasculogenic properties are restricted to some populations of MSCs or whether the entire population of MSCs has these properties. We cultured two different populations of MSCs in different culture media and their vasculogenic properties were evaluated using In vitro spheroid sprouting assay. Neither population of MSCs expressed markers of endothelial progenitor cells (EPCs), but they were different in the profiling of angiogenic factor expression as well as vasculogenic properties. One population of MSCs expressed basic fibroblast growth factor (bFGF) and another expressed hepatocyte growth factor (HGF). MSCs expressing HGF exhibited In vitro angiogenic sprouting capacity in response to bFGF derived from other MSCs as well as to their autocrine HGF. The vasculogenic mesenchymal stem cells (vMSCs) derived from the bone marrow also enhanced In vitro angiogenic sprouting capacity of human umbilical vein endothelial cells (HUVECs) in an HGF-dependent manner. These results suggest that MSCs exhibit different vasculogenic properties, and vMSCs that are different from EPCs may contribute to neovascularization and could be a promising cellular therapy for cardiovascular diseases.

## 1. Introduction

Endothelial progenitor cells (EPCs) were first identified from mononuclear cells, expressing CD34, in peripheral blood and were shown to contribute to the formation of new blood vessels in an animal model of hind limb ischemia [1]. Subsequently, two different types of EPCs were identified upon In vitro culture of peripheral blood mononuclear cells [2,3,4,5]. EPCs that developed colonies consisting of spindle-shaped cells early in culture (4–7 days) expressed CD34 and CD133. They showed limited proliferative capacity and started to disappear after culturing for 4–6 weeks. Another type of EPCs, late EPCs, were also identified after prolonged culturing with vascular endothelial growth factor (VEGF). After 2–3 weeks of culturing, these cells developed colonies with cobblestone appearance and were able to proliferate up to 8 weeks after culturing. Late EPCs express CD34 and are derived from mononuclear cells mobilized from the bone marrow [4,6]. In addition to peripheral blood mononuclear cells, EPCs were cultured from mononuclear cells purified from the bone marrow [7,8,9]. Multipotent non-endothelial stromal cells expressing neither CD34 nor VE-cadherin were identified from postnatal bone marrow. They first differentiated into cells expressing CD34 and VE-cadherin In vitro, in response to VEGF treatment, and eventually differentiated into endothelial cells and were called EPCs [8]. Although cell surface markers have not yet been identified for detecting EPCs and determining their origin, interest in the therapeutic effects of EPCs in cardiovascular disease has been growing.

EPCs are known to contribute to increased neovascularization through vasculogenesis and angiogenesis [10]. Transplantation of EPCs enhanced neovascular formation in ischemic tissues of adult animals following their incorporation into blood vessels and their differentiation into mature endothelial cells [1,10,11,12]. Several studies have suggested that EPC-initiated angiogenesis is a major contributor to neovascularization [10,13,14]. EPCs mediate angiogenesis by secreting paracrine factors, including proangiogenic cytokines and growth factors such as VEGF, stromal cell-derived factor 1 (SDF-1), and hepatocyte growth factor (HGF) [10,15]. EPCs enhance the survival of mature endothelial cells, undergoing oxidative stress, by providing paracrine factors such as VEGF and HGF to these cells [16]. EPCs also increase the migration of endothelial cells and mesenchymal stem cells (MSCs) by secreting SDF-1 [15].

Mesenchymal stem cells derived from human bone marrow are a rare population of stromal cells that have multipotent capacity and self-renewing potential. MSCs are also known to contribute to vascularization [17,18,19,20]. MSCs function as perivascular cells in vivo [18,19]. In vitro, MSCs differentiate into endothelial cells [17] and form capillary tube-like structures [20]. However, it remains unclear whether the vasculogenic capacity is heterogeneous in MSC populations.

Several studies have suggested that the interaction between EPCs and MSCs contribute to promoting the proliferation of each other and neovascularization through paracrine factors as well as through direct adhesion [21,22,23,24]. MSCs adhere to capillary-like tubes formed by EPCs under In vitro angiogenic experimental conditions, and their self-renewal capacity is enhanced by EPCs [21]. MSCs increase EPCs proliferation by secreting insulin-like growth factor-1 [22] and reverse the senescent phenotype of EPCs In vitro [23]. MSCs induce the differentiation of EPCs into endothelial cells by secreting VEGF [24]. However, the precise mechanism underlying these interactions remains unclear.

In this study, we examined whether the vasculogenic capacity is heterogeneous in MSC populations and also investigated whether the different types of MSCs functionally interact to enhance vasculogenesis. Two different types of MSCs were cultured from human bone marrow mononuclear cells under different culture conditions. One population of MSCs was cultured using a general culture medium developed for culturing MSCs. Another population of MSCs was cultured using endothelial cell culture medium containing VEGF without any cell sorting process. Since this population of MSCs showed angiogenic sprouting capacity in response to basic fibroblast growth factor (bFGF), one of the important angiogenic factors, in contrast to the first population of MSCs, these cells were named vasculogenic MSCs (vMSCs) in the current study. Neither MSCs nor vMSCs expressed the markers (CD34, CD133, and VE-cadherin) of EPCs that have been identified by other studies. We demonstrated that MSCs increased the angiogenic sprouting capacity of vMSCs via bFGF. Furthermore, vMSCs increased the angiogenic sprouting of human umbilical vein endothelial cells (HUVECs) by secreting hepatocyte growth factor (HGF), a paracrine factor. These results suggest that MSCs exhibit different vasculogenic properties and that vMSCs may contribute to vasculogenesis via their sprouting capacity in response to both bFGF and HGF. In addition, they increase angiogenesis via the paracrine effects of HGF in mature endothelial cells.

## 2. Results

### 2.1. Increase in Angiogenic Sprouting Capacity of vMSCs in Response to bFGF and HGF

**Figure 1:**
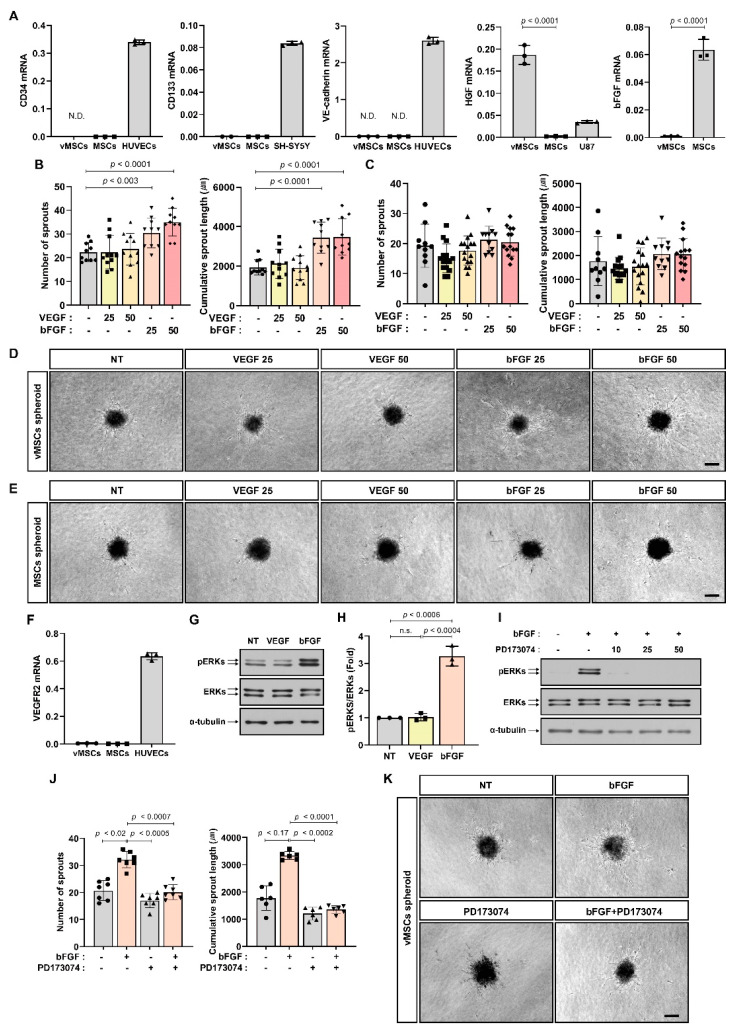
In vitro angiogenic sprouting of vMSCs in response to bFGF. (**A**) qRT-PCR analysis of mRNA expression of CD34, CD133, VE-cadherin, HGF, and bFGF in vMSCs and MSCs. HUVECs, SH-SY5Y, and U87 cells were used as positive controls that are known to express each gene. ND; not detected. (**B**–**E**) Sprouting capacity of vMSC spheroids and MSC spheroids incubated with VEGF (25 ng/mL or 50 ng/mL) and bFGF (25 ng/mL or 50 ng/mL). Representative images are shown in (**D**,**E**). (**F**) qRT-PCR analysis of mRNA expression of VEGFR2 in vMSCs and MSCs. HUVECs were used as positive controls that are known to express VEGFR2. (**G**,**H**) Western blot analysis of phosphorylated ERKs (pERKs) and ERKs in vMSCs treated with VEGF (25 ng/mL) and bFGF (25 ng/mL) for 10 min. α-Tubulin was used as an internal control. (**H**) Densitometric analysis of Western blot results (three independent experiments). (**I**) Western blot analysis of phosphorylated ERKs (pERKs) and ERKs in vMSCs pretreated with PD173074 (10 nM, 25 nM, or 50 nM) for 3 h followed by treatment with bFGF (25 ng/mL) for 10 min (two independent experiments). α-Tubulin was used as an internal control. (**J**,**K**) Sprouting capacity of vMSC spheroids pretreated with PD173074 (10 nM) for 3 h followed by treatment with bFGF (25 ng/mL) for 48 h. Results are presented as the mean ± SD. n.s.; not significant. Scale bar; 100 μm. Uncropped Western blot images are shown in Figure S1.

MSCs and vMSCs expressed CD29, CD90, CD73, and CD105 that are the surface markers of MSCs [25,26], but they did not express CD34 and CD133, both of which have been known to be surface markers of EPCs [1,2] (Figure 1A). VE-cadherin, a marker of mature endothelial cells, was not expressed in either MSCs or vMSCs (Figure 1A). Both HGF and bFGF have been demonstrated to be positively involved in angiogenesis and are secreted by mesenchymal cells [27,28]. vMSCs expressed higher levels of HGF than MSCs; however, MSCs expressed higher levels of bFGF than vMSCs (Figure 1A). These results suggest that MSCs and vMSCs are different cell populations, although they were cultured from bone marrow mononuclear cells and showed similar surface marker expression pattern. Then, we examined In vitro angiogenic capacity of MSCs and vMSCs in response to angiogenic factors by performing In vitro spheroid sprouting assay using spheroids of MSCs and vMSCs. MSC spheroids and vMSC spheroids did not show difference in angiogenic sprouting ability in the absence of stimulation by angiogenic factors (Figure 1B–E). MSCs and vMSCs did not respond to VEGF, which is the most important angiogenic factor (Figure 1B–E). These results were consistent with the observation that MSCs and vMSCs did not express VEGF receptor (Figure 1F). Next, the effect of bFGF on angiogenic sprouting of MSCs and vMSCs was investigated. vMSC spheroids increased angiogenic sprouting ability in response to bFGF, which was expressed in MSCs, but MSC spheroids did not increase angiogenic sprouting (Figure 1B–E). Extracellular signal-regulated kinases (ERKs) are major signal transducers of both bFGF and VEGF, and ERKs are phosphorylated upon activation of their receptors [29]. ERK phosphorylation was increased in vMSCs treated with bFGF, but not in those treated with VEGF (Figure 1G,H). PD173074, an inhibitor of bFGF receptor [30] impaired bFGF-mediated increase in angiogenic sprouting ability of vMSCs as well as ERK phosphorylation in vMSCs treated with bFGF (Figure 1I–K).

**Figure 2:**
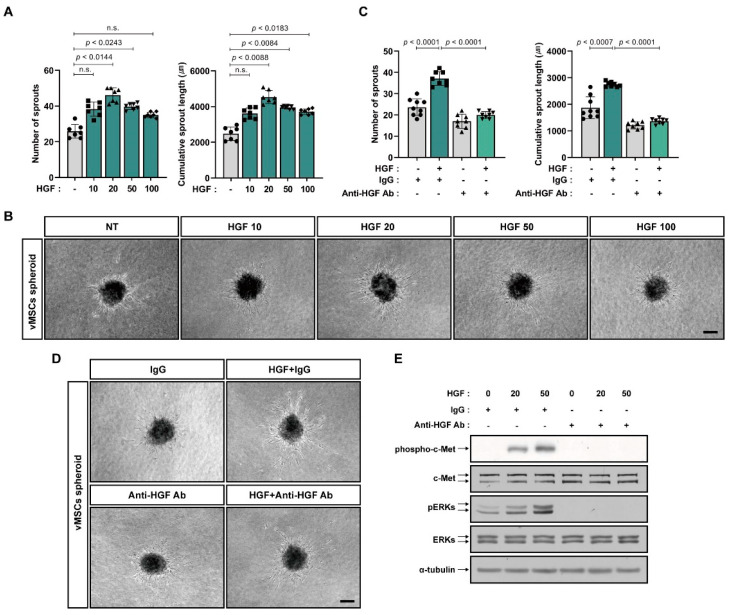
In vitro angiogenic sprouting of vMSCs in response to HGF. (**A**,**B**) Sprouting capacity of vMSC spheroids incubated with HGF (10, 20, 50 or 100 ng/mL). Representative images are shown in (**B**). (**C**,**D**) Sprouting capacity of vMSC spheroids treated with HGF (20 ng/mL) and/or HGF neutralizing antibody (anti-HGF Ab, 500 ng/mL) for 48 h. Mouse IgG (IgG, 500 ng/mL) was used as the control. Representative images are shown in (**D**). (**E**) Western blot analysis of phosphorylated c-Met (phospho-c-Met), c-Met, phosphorylated ERKs (pERKs), and ERKs in vMSCs treated with HGF (20 ng/mL or 50 ng/mL) and/or HGF neutralizing antibody (anti-HGF Ab, 500 ng/mL) for 5 min. Mouse IgG (IgG, 500 ng/mL) was used as the control. α-Tubulin was used as an internal control. Results are presented as the mean ± SD. n.s.; not significant. Scale bar; 100 μm. Uncropped Western blot images are shown in Figure S2.

It has been shown that HGF protected EPCs from senescence and enhanced proangiogenic property of EPCs [31]. The In vitro angiogenic sprouting capacity of vMSCs increased in response to HGF treatment (Figure 2A,B). HGF phosphorylates c-Met, HGF receptor and several intracellular proteins, including ERKs to intrinsically transduce its signal. The HGF neutralizing antibody impaired HGF-induced increase in the sprouting capacity of vMSCs (Figure 2C,D) as well as HGF-induced phosphorylation of c-Met and ERK in vMSCs (Figure 2E).

### 2.2. Increase in Angiogenic Sprouting Capacity of vMSCs in Response to Paracrine Activity of vMSCs and MSCs

**Figure 3:**
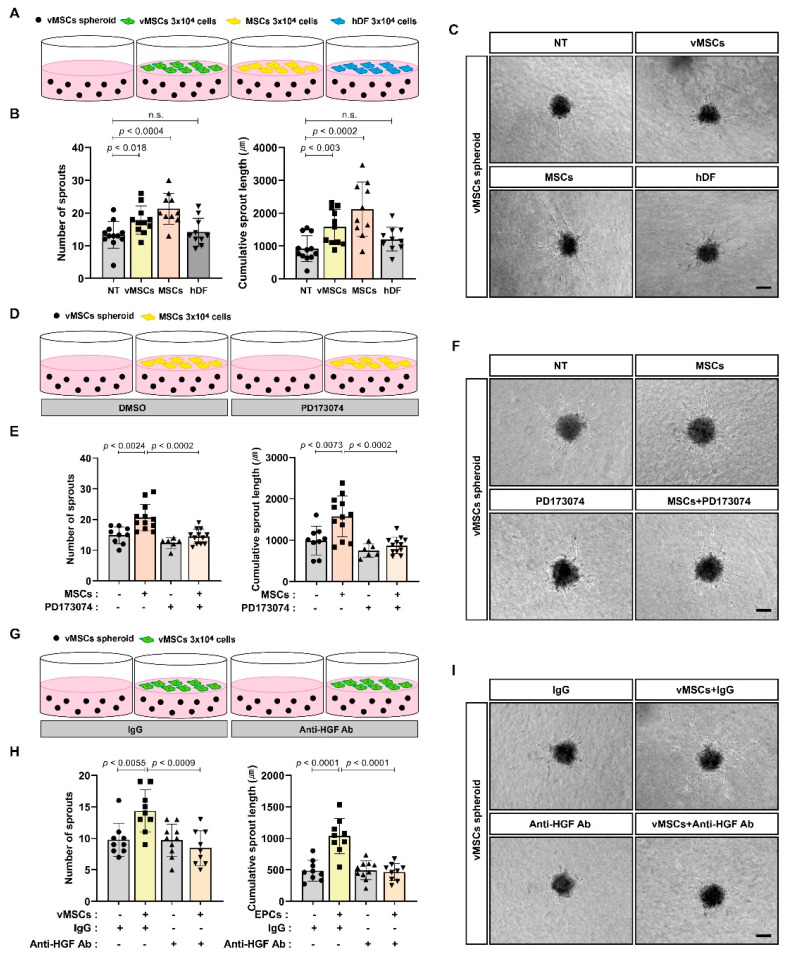
In vitro angiogenic sprouting of vMSCs in response to paracrine factors derived from vMSCs and MSCs. (**A**–**C**) Sprouting capacity of vMSC spheroids co-cultured with vMSCs, MSCs or hDF for 48 h. Schematic representation in (**A**) and representative images in (**C**). (**D**–**F**) Sprouting capacity of vMSC spheroids pretreated with PD173074 (10 nM) for 3 h followed by co-culture with MSCs for 48 h. Schematic representation in (**D**) and representative images in (**F**). (**G**–**I**) Sprouting capacity of vMSC spheroids pretreated with vMSCs and/or HGF neutralizing antibody (anti-HGF Ab, 500 ng/mL) for 48 h. Mouse IgG (IgG, 500 ng/mL) was used as the control. Schematic representation in (**G**) and representative images in (**I**). Results are presented as the mean ± SD. n.s.; not significant. Scale bar; 100 μm.

Interactions between MSCs and EPCs play important roles in their proangiogenic function, and the paracrine activity of MSCs and EPCs may be essential for their angiogenic capacity. We examined whether MSCs can regulate the sprouting capacity of vMSCs by providing paracrine factors, including bFGF, expressed by MSCs and whether vMSCs are able to control their own sprouting capacity by secreting angiogenic factors such as HGF. We performed In vitro spheroid sprouting assay using vMSC spheroids by co-culturing them with MSCs or vMSCs (Figure 3A). Both MSCs and vMSCs enhanced the sprouting capacity of vMSC spheroids in contrast to dermal fibroblasts (hDF), used as the control (Figure 3B,C). PD173074 and HGF neutralizing antibody impaired MSCs-induced increase in the sprouting capacity of vMSCs (Figure 3D–I).

### 2.3. Increase in Angiogenic Sprouting Capacity of HUVECs in Response to vMSCs-Derived HGF

**Figure 4:**
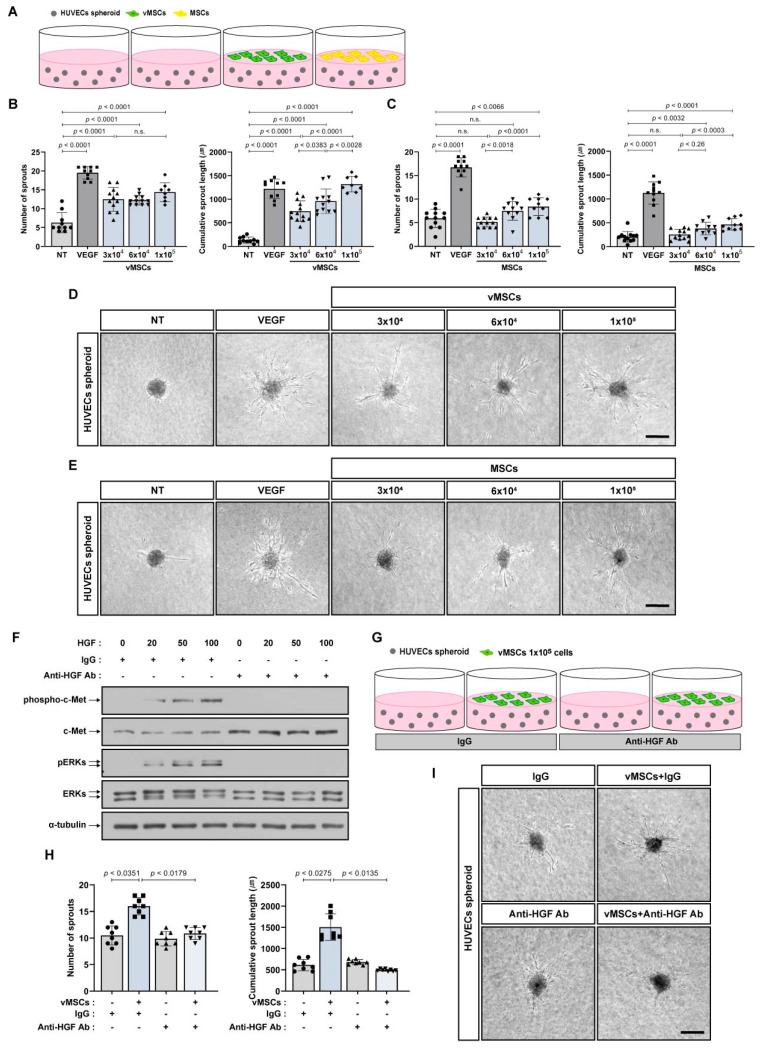
In vitro angiogenic sprouting of HUVEC spheroids in response to vMSCs-derived HGF. (**A**–**E**) Sprouting capacity of HUVEC spheroids co-cultured with vMSCs or MSCs for 48 h. VEGF was used as the positive control that enhances angiogenic sprouting of HUVEC spheroids. Schematic representation in (**A**) and representative images in (**D**,**E**). (**F**) Western blot analysis of phosphorylated c-Met (phospho-c-Met), c-Met, phosphorylated ERKs (pERKs), and ERKs in HUVECs treated with HGF (20, 50 or 100 ng/mL) and/or HGF neutralizing antibody (anti-HGF Ab, 500 ng/mL) for 5 min. Mouse IgG (IgG, 500 ng/mL) was used as the control. α-Tubulin was used as an internal control. (**G**–**I**) Sprouting capacity of HUVEC spheroids co-cultured with vMSCs for 48 h. Cells were treated with HGF neutralizing antibody (anti-HGF Ab) or mouse IgG (IgG) at a concentration of 500 ng/mL. Schematic representation in (**G**) and representative images in (**I**). Results are presented as the mean ± SD. n.s.; not significant. Scale bar; 100 μm. Uncropped Western blot images are shown in Figure S3.

It has been suggested that EPCs provide proangiogenic benefits to mature endothelial cells [21,22,23,24]. We examined the proangiogenic effects of vMSCs on HUVECs. vMSCs increased the In vitro sprouting capacity of HUVECs similar to VEGF-induced increase in the sprouting capacity of HUVECs, In vitro. However, MSCs showed a slightly higher increase. (Figure 4A–D). Next, we investigated the mechanisms by which vMSCs enhanced the angiogenic sprouting of HUVECs. In contrast to MSCs, vMSCs expressed HGF (Figure 1A), and it has been reported that HGF contributes to increased angiogenesis and survival of HUVECs [32]. HGF increased the phosphorylation of both c-Met and ERKs in HUVECs, and HGF neutralizing antibody impaired their phosphorylation induced by HGF (Figure 4F). Moreover, HGF neutralizing antibody inhibited vMSC-induced increase in the sprouting capacity of HUVECs (Figure 4G–I).

## 3. Discussion

vMSCs cultured in endothelial cell culture medium supplemented with VEGF showed increased In vitro angiogenic sprouting in response to bone marrow MSCs-derived essential angiogenic growth factor, bFGF, as well as in response to their own paracrine factor, HGF. Moreover, vMSCs enhanced In vitro angiogenic sprouting of HUVECs in an HGF-dependent manner.

Both vMSCs and MSCs were derived from bone marrow mononuclear cells and showed common characteristics such as negative expression of CD34, CD133, and VE-cadherin. Both types of cells did not enhance their own In vitro angiogenic sprouting capacity in response to the most potent angiogenic factor, VEGF. However, they differ in several characteristics related to neovascularization. vMSCs were able to increase In vitro angiogenic sprouting capacity in response to bFGF, but MSCs could not. In vitro angiogenic sprouting of HUVEC spheroids significantly increased in response to vMSCs but not in response to MSCs. Moreover, vMSCs expressed HGF mRNA but not bFGF mRNA. However, the expression of HGF and bFGF in MSCs was the opposite of that in vMSCs. These results suggest that bone marrow vMSCs are different from MSCs derived from the bone marrow. Unfortunately, no markers have been identified for detecting vMSCs. Therefore, further experiments are required to identify markers that can be used to distinguish vMSCs from MSCs and detect vMSCs.

The current study suggests that there may be different types of mesenchymal stem cells in adult bone marrow. Various essential roles of mesenchymal stem cells in regeneration have been demonstrated, although they are a rare cell population in the bone marrow [33]. They have been differentiated under In vitro culture conditions into adipocytes, osteoblasts, and chondrocytes [33]. These cells secrete anti-inflammatory cytokines and immunomodulatory paracrine factors [34]. It also has been shown that MSCs play important roles in neovascularization. MSCs regulate angiogenesis and vasculogenesis through providing proangiogenic paracrine factors to existing mature endothelial cells as well as to EPCs [35]. MSCs directly differentiate into various vascular cells such as endothelial cells, pericytes, and smooth muscle cells [17,35,36]. These results suggest that mesenchymal stem cells of adult bone marrow may be heterogeneous in their vascular regeneration capacity. The current study suggested that two different types of the bone marrow-derived mesenchymal stem cells, vMSCs and MSCs may differently contribute to vasculogenesis. However, it remains unclear whether the heterogeneous characteristics of mesenchymal stem cells derived from the bone marrow originate from clonal diversity or are regulated by extracellular extrinsic factors. It would be interesting and important to examine whether vMSCs and MSCs may reversibly interchange.

EPCs have been shown to contribute to neovascularization of ischemic tissues and regeneration of injured blood vessels. Therefore, cell therapy using EPCs is a promising strategy for cardiovascular regeneration. EPCs have been isolated from various sources, including peripheral blood and bone marrow. However, the identification and culture conditions of EPCs are still out of consensus. The problems associated with EPCs make them difficult to be used in clinical trials. The current study suggests that vMSCs may be a potential source for future cell-based vascular therapies, although vMSCs differ from EPCs. Further experiments are required to prove the possibility that vMSCs may be used for cardiovascular regeneration instead of EPCs, which still has problems that need to be solved for use in clinical trials.

## 4. Materials and Methods

### 4.1. Cell Culture

Human bone marrow mononuclear cells (BM-MNCs) from healthy adult donors aged between 27 and 39 years were purchased from LONZA (Basel, Switzerland). BM-MNCs were thawed and resuspended using the StemMACS MSC Expansion media kit XF (Miltenyi Biotec, Bergisch Gladbach, Germany). After centrifugation at 430× *g* for 10 min at 25°C, the cells were seeded at a density of 1.3 × 10^5^ cells/cm^2^ in a culture flask coated with humatein (100 μg/mL, ROKIT healthcare, Seoul, Republic of Korea) and maintained using fetal bovine serum (FBS)-depleted EGM-2 bulletkit medium (Lonza; Basel, Switzerland) supplemented with 2% human platelet lysate (PL bioscience GmbH, Germany) and 1 KU heparin (Sigma-Aldrich, St. Louis, MO, USA) to obtain vMSCs. The culture medium was changed every day for 7 days, and the cells were maintained for further 2 to 4 days. To culture MSCs, BM-MNCs were seeded at a density of 4 × 10^4^ cells/cm^2^ using the StemMACS MSC Expansion media kit XF [37,38], and the medium was changed the next day. The cells were maintained for 9 to 10 days without changing the medium. MSCs and vMSCs at passage 3 were used for all experiments. Human umbilical vein endothelial cells (HUVECs; Lonza) were cultured in culture dishes coated with 2% bovine skin gelatin (Sigma-Aldrich) using the Endothelial Cell Growth Media-Plus bulletkit (EGM-Plus; Lonza) [39]. HUVECs between passages 4 and 8 were used for all experiments. Human dermal fibroblasts (hDF) were obtained from LONZA and maintained in Dulbecco’s modified Eagle medium (DMEM), containing 5.5 mM glucose (GE Healthcare Life Sciences, Logan, UT, USA) supplemented with 10% FBS (Invitrogen, Waltham, MA, USA) and 1% penicillin and streptomycin (Invitrogen). All the cells were maintained at 37 °C in a humidified incubator containing 5% CO_2_.

### 4.2. Real-Time Polymerase Chain Reaction (RT-PCR)

Total RNA was isolated using TRIzol reagent (Invitrogen), and cDNA was synthesized using SuperScript III Reverse Transcriptase (Invitrogen) according to the manufacturer’s protocol. RT-PCR was performed using SYBR Green reagent (Invitrogen) according to standard procedures [40]. Human ribosomal protein S9 (RPS9) was used as the endogenous control. Primer sequences were as follows: CD34 (forward):5′-GAGTTTGCTGCCTTCTGGGTTC-3′, CD34 (reverse):5′-GCAGGCTGGTACTTCCAAGG-3′; CD133 (forward):5′-GCAACAGCGATCAAGGAGAC-3′, CD133 (reverse):5′-CACCAAGCACAGAGGGTCAT-3′; CDH5 (cadherin 5) (forward):5′-CTTCACCCAGACCAAGTACACA-3′, CDH5 (reverse):5′-GGCTCATGTATCGGAGGTCG-3′; HGF (forward):5′-CGGGGTAAAGACCTACAGGA-3′, HGF (reverse):5′-CTGAGGAATGTCACAGACTTCG-3′; FGF2 (forward):5′-CGACCCTCACATCAAGCTAC-3′, FGF2 (reverse):5′-CAGTGCCACATACCAACTGG-3′; KDR (forward):5′-GCTGAAGCTAGGTAAGCCTC-3′, KDR (reverse):5′-CATGAGAGCTCGATGCTCACTG-3′; RPS9 (forward):5′-CTGACGCTTGATGAGAAGGAC-3,’ RPS9 (reverse):5′-CAGCTTCATCTTGCCCTCAT-3′.

### 4.3. In Vitro Spheroid Sprouting Assay

In vitro spheroid sprouting assay was performed using standard procedures [41]. MSCs and vMSCs were resuspended at a density of 2 × 10^4^ cells/mL in Medium 199 (Sigma-Aldrich) containing 10% FBS (Thermo Fisher Scientific, Waltham, MA, USA) and 0.2% methylcellulose (Sigma-Aldrich), and HUVECs were resuspended at a density of 1.33 × 10^4^ cells/mL. The cell suspension (30 µL) was seeded on the non-adherent lid of a Petri dish and cultured for 24 h to form spheroids. The spheroids were washed with phosphate-buffered saline (PBS) and harvested by centrifugation (200× *g* for 5 min at 25 °C). MSC and vMSC spheroids were resuspended in Medium 199 supplemented with 0.95% methylcellulose, and HUVEC spheroids were resuspended in Medium 199 supplemented with 20% FBS and 0.95% methylcellulose. Type I collagen solution was prepared on ice as a mixture (8:1:1) of 3 mg/mL type I-A collagen (Nitta Gelatin), 10× Medium 199 (Sigma-Aldrich), and 10× reconstitution buffer (0.05 N NaOH, 0.261 M NaHCO_3_, and 0.2 M HEPES). The resuspended spheroids were mixed at a ratio of 1:1 on ice with the prepared type I collagen solution and transferred into wells of 24 well plates (0.7 mL/well). After incubating for 30 min to polymerize the gel, 100 μL of Medium 199 was added on top of the gel and further incubated for 24 h or 48 h. To analyze the angiogenic effects of VEGF, bFGF, and HGF on spheroids, they were stimulated with Medium 199 containing 20 ng/mL VEGF_165_ (Peprotech, Cranbury, NJ, USA), bFGF (R&D Systems, Minneapolis, MN, USA), or HGF (Peprotech). If necessary, the spheroids were pretreated with PD173074 (Tocris, Bristol, UK) or HGF neutralizing antibody (MAB294, R&D Systems) for 3 h prior to treatment with bFGF or HGF, respectively. To examine the effect of MSCs and vMSCs on spheroid sprouting, MSCs or vMSCs were resuspended in Medium 199, and the cell suspension (100 μL) was added on top of the type I collagen gel containing spheroids. The number of sprouts and the length of all sprouts per spheroid (cumulative sprout length) were measured using ImageJ software (NIH, Bethesda, MD, USA).

### 4.4. Western Blot Analysis

vMSCs cultured in their own culture medium were starved overnight with Medium 199 supplemented with 2% FBS. The vMSCs were then treated with VEGF_165_, bFGF, or HGF. If required, cells were pretreated with PD173074 for 3 h prior to treatment with bFGF, as well as co-treated with HGF neutralizing antibody and HGF. HUVECs were seeded on culture plates coated with bovine plasma fibronectin (10 μg/mL, Sigma-Aldrich) and cultured in their own culture medium. HUVECs were then starved with Endothelial Cell Basal Medium-Plus (Lonza) supplemented with 1% bovine serum albumin (Sigma-Aldrich). After starvation for 6 h, HUVECs were treated with HGF. If necessary, HUVECs were co-treated with HGF-neutralizing antibody and HGF. The cells were rinsed twice with ice-cold PBS and lysed using 2x SDS buffer (100 mM Tris-HCl, pH 6.8, 20% glycerol, 2% sodium dodecyl sulfate (SDS), 0.001% bromophenol blue, and 10% β-mercaptoethanol) at 25 °C for 5 min. Cell lysates were collected by scraping and denatured by heating at 95 °C for 5 min. Western blotting of proteins in the cell lysate was performed using standard procedures [42]. Primary antibodies against anti-phospho-ERKs (pERKs; 1:3000; Cell Signaling Technology, Danvers, MA, USA), anti-ERKs (1:3000; Cell Signaling Technology), anti-phospho-c-Met (pc-Met; 1:1000; Cell Signaling Technology), anti-c-Met (1:1000; Cell Signaling Technology), and α-tubulin (1:30,000–1:50,000, Sigma-Aldrich) were used for immunoblotting. If necessary, band densities were measured using the ImageJ software (NIH).

### 4.5. Statistical Analysis

Quantitative data are presented as mean ± standard deviation (SD). Statistical analysis was performed using Student’s *t*-test using GraphPad version 9.4.0 (GraphPad Software Inc., CA, USA; http://www.graphpad.com). Statistical significance was set at *p* < 0.05.

## Data Availability

The data supporting the findings of this study are available from the corresponding author upon reasonable request.

## Supplementary Materials

The following supporting information can be downloaded at: https://www.mdpi.com/article/10.3390/ijms24010413/s1.

## References

[1] Asahara T., Murohara T., Sullivan A., Silver M., van der Zee R., Li T., Witzenbichler B., Schatteman G., Isner J.M. (1997). Isolation of putative progenitor endothelial cells for angiogenesis. Science.

[2] Yan F., Liu X., Ding H., Zhang W. (2022). Paracrine mechanisms of endothelial progenitor cells in vascular repair. Acta Histochem..

[3] Hur J., Yoon C.H., Kim H.S., Choi J.H., Kang H.J., Hwang K.K., Oh B.H., Lee M.M., Park Y.B. (2004). Characterization of two types of endothelial progenitor cells and their different contributions to neovasculogenesis. Arter. Thromb. Vasc. Biol..

[4] Shi Q., Rafii S., Wu M.H., Wijelath E.S., Yu C., Ishida A., Fujita Y., Kothari S., Mohle R., Sauvage L.R. (1998). Evidence for circulating bone marrow-derived endothelial cells. Blood.

[5] Medina R.J., O’Neill C.L., O’Doherty T.M., Wilson S.E., Stitt A.W. (2012). Endothelial progenitors as tools to study vascular disease. Stem. Cells Int..

[6] Lin Y., Weisdorf D.J., Solovey A., Hebbel R.P. (2000). Origins of circulating endothelial cells and endothelial outgrowth from blood. J. Clin. Investig..

[7] Brandl A., Yuan Q., Boos A.M., Beier J.P., Arkudas A., Kneser U., Horch R.E., Bleiziffer O. (2014). A novel early precursor cell population from rat bone marrow promotes angiogenesis in vitro. BMC Cell Biol..

[8] Reyes M., Dudek A., Jahagirdar B., Koodie L., Marker P.H., Verfaillie C.M. (2002). Origin of endothelial progenitors in human postnatal bone marrow. J. Clin. Investig..

[9] Sekiguchi H., Ii M., Jujo K., Yokoyama A., Hagiwara N., Asahara T. (2011). Improved culture-based isolation of differentiating endothelial progenitor cells from mouse bone marrow mononuclear cells. PLoS ONE.

[10] Krenning G., van Luyn M.J., Harmsen M.C. (2009). Endothelial progenitor cell-based neovascularization: Implications for therapy. Trends Mol. Med..

[11] Murohara T., Ikeda H., Duan J., Shintani S., Sasaki K., Eguchi H., Onitsuka I., Matsui K., Imaizumi T. (2000). Transplanted cord blood-derived endothelial precursor cells augment postnatal neovascularization. J. Clin. Investig..

[12] Kalka C., Masuda H., Takahashi T., Kalka-Moll W.M., Silver M., Kearney M., Li T., Isner J.M., Asahara T. (2000). Transplantation of ex vivo expanded endothelial progenitor cells for therapeutic neovascularization. Proc. Natl. Acad. Sci. USA.

[13] Heil M., Ziegelhoeffer T., Mees B., Schaper W. (2004). A different outlook on the role of bone marrow stem cells in vascular growth: Bone marrow delivers software not hardware. Circ. Res..

[14] Kinnaird T., Stabile E., Burnett M.S., Lee C.W., Barr S., Fuchs S., Epstein S.E. (2004). Marrow-derived stromal cells express genes encoding a broad spectrum of arteriogenic cytokines and promote in vitro and in vivo arteriogenesis through paracrine mechanisms. Circ. Res..

[15] Tamari T., Kawar-Jaraisy R., Doppelt O., Giladi B., Sabbah N., Zigdon-Giladi H. (2020). The Paracrine Role of Endothelial Cells in Bone Formation via CXCR4/SDF-1 Pathway. Cells.

[16] Yang Z., von Ballmoos M.W., Faessler D., Voelzmann J., Ortmann J., Diehm N., Kalka-Moll W., Baumgartner I., Di Santo S., Kalka C. (2010). Paracrine factors secreted by endothelial progenitor cells prevent oxidative stress-induced apoptosis of mature endothelial cells. Atherosclerosis.

[17] Oswald J., Boxberger S., Jorgensen B., Feldmann S., Ehninger G., Bornhauser M., Werner C. (2004). Mesenchymal stem cells can be differentiated into endothelial cells in vitro. Stem. Cells.

[18] Crisan M., Yap S., Casteilla L., Chen C.W., Corselli M., Park T.S., Andriolo G., Sun B., Zheng B., Zhang L. (2008). A perivascular origin for mesenchymal stem cells in multiple human organs. Cell Stem. Cell.

[19] Shi S., Gronthos S. (2003). Perivascular niche of postnatal mesenchymal stem cells in human bone marrow and dental pulp. J. Bone Min. Res..

[20] Du W.J., Chi Y., Yang Z.X., Li Z.J., Cui J.J., Song B.Q., Li X., Yang S.G., Han Z.B., Han Z.C. (2016). Heterogeneity of proangiogenic features in mesenchymal stem cells derived from bone marrow, adipose tissue, umbilical cord, and placenta. Stem. Cell Res..

[21] Zhang H., Xian L., Lin Z., Yang C., Zhang M., Feng W., Peng X., Chen X., Wu X. (2014). Endothelial progenitor cells as a possible component of stem cell niche to promote self-renewal of mesenchymal stem cells. Mol. Cell. Biochem..

[22] Hou J., Peng X., Wang J., Zhang H., Xia J., Ge Q., Wang X., Chen X., Wu X. (2017). Mesenchymal stem cells promote endothelial progenitor cell proliferation by secreting insulinlike growth factor1. Mol. Med. Rep..

[23] Wang L., Wei J., Da Fonseca Ferreira A., Wang H., Zhang L., Zhang Q., Bellio M.A., Chu X.M., Khan A., Jayaweera D. (2020). Rejuvenation of Senescent Endothelial Progenitor Cells by Extracellular Vesicles Derived from Mesenchymal Stromal Cells. JACC Basic Transl. Sci..

[24] Ge Q., Zhang H., Hou J., Wan L., Cheng W., Wang X., Dong D., Chen C., Xia J., Guo J. (2018). VEGF secreted by mesenchymal stem cells mediates the differentiation of endothelial progenitor cells into endothelial cells via paracrine mechanisms. Mol. Med. Rep..

[25] Fathi E., Azarbad S., Farahzadi R., Javanmardi S., Vietor I. (2022). Effect of Rat Bone Marrow Derived-Mesenchymal Stem Cells on Granulocyte Differentiation of Mononuclear Cells as Preclinical Agent in Cellbased Therapy. Curr. Gene.

[26] Lv F.J., Tuan R.S., Cheung K.M., Leung V.Y. (2014). Concise review: The surface markers and identity of human mesenchymal stem cells. Stem. Cells.

[27] Matsumoto K., Nakamura T. (1996). Emerging multipotent aspects of hepatocyte growth factor. J. Biochem..

[28] Folkman J., Shing Y. (1992). Angiogenesis. J. Biol. Chem..

[29] Ornitz D.M., Itoh N. (2015). The Fibroblast Growth Factor signaling pathway. Wiley Interdiscip. Rev. Dev. Biol..

[30] Mohammadi M., Froum S., Hamby J.M., Schroeder M.C., Panek R.L., Lu G.H., Eliseenkova A.V., Green D., Schlessinger J., Hubbard S.R. (1998). Crystal structure of an angiogenesis inhibitor bound to the FGF receptor tyrosine kinase domain. EMBO J..

[31] Schroder K., Schutz S., Schloffel I., Batz S., Takac I., Weissmann N., Michaelis U.R., Koyanagi M., Brandes R.P. (2011). Hepatocyte growth factor induces a proangiogenic phenotype and mobilizes endothelial progenitor cells by activating Nox2. Antioxid. Redox Signal.

[32] Xin X., Yang S., Ingle G., Zlot C., Rangell L., Kowalski J., Schwall R., Ferrara N., Gerritsen M.E. (2001). Hepatocyte growth factor enhances vascular endothelial growth factor-induced angiogenesis in vitro and in vivo. Am. J. Pathol..

[33] Prockop D.J. (1997). Marrow stromal cells as stem cells for nonhematopoietic tissues. Science.

[34] Han Y., Yang J., Fang J., Zhou Y., Candi E., Wang J., Hua D., Shao C., Shi Y. (2022). The secretion profile of mesenchymal stem cells and potential applications in treating human diseases. Signal Transduct. Target.

[35] Shi W., Xin Q., Yuan R., Yuan Y., Cong W., Chen K. (2021). Neovascularization: The Main Mechanism of MSCs in Ischemic Heart Disease Therapy. Front. Cardiovasc. Med..

[36] Abedin M., Tintut Y., Demer L.L. (2004). Mesenchymal stem cells and the artery wall. Circ. Res..

[37] Schmelzer E., McKeel D.T., Gerlach J.C. (2019). Characterization of Human Mesenchymal Stem Cells from Different Tissues and Their Membrane Encasement for Prospective Transplantation Therapies. Biomed Res. Int..

[38] Clavreul A., Pourbaghi-Masouleh M., Roger E., Lautram N., Montero-Menei C.N., Menei P. (2017). Human mesenchymal stromal cells as cellular drug-delivery vectors for glioblastoma therapy: A good deal?. J. Exp. Clin. Cancer Res..

[39] Park K.S., Schecterson L., Gumbiner B.M. (2021). Enhanced endothelial barrier function by monoclonal antibody activation of vascular endothelial cadherin. Am. J. Physiol. Heart Circ. Physiol..

[40] Fathi E., Vandghanooni S., Montazersaheb S., Farahzadi R. (2021). Mesenchymal stem cells promote caspase-3 expression of SH-SY5Y neuroblastoma cells via reducing telomerase activity and telomere length. Iran. J. Basic Med. Sci..

[41] Kim W., Park A., Jang H.H., Kim S.E., Park K.S. (2022). Breast Tumor Cell-Stimulated Bone Marrow-Derived Mesenchymal Stem Cells Promote the Sprouting Capacity of Endothelial Cells by Promoting VEGF Expression, Mediated in Part through HIF-1alpha Increase. Cancers.

[42] Dubon M.J., Yu J., Choi S., Park K.S. (2018). Transforming growth factor beta induces bone marrow mesenchymal stem cell migration via noncanonical signals and N-cadherin. J. Cell. Physiol..

