# Supplementary material for: Bone Marrow-Derived Vasculogenic Mesenchymal Stem Cells Enhance In Vitro Angiogenic Sprouting of Human Umbilical Vein Endothelial Cells
Source: Int J Mol Sci. 2022 Dec 27;24(1):413. doi: 10.3390/ijms24010413 (PMC9820660; doi:10.3390/ijms24010413)
Supplement: Supplementary file 1 [file ijms-24-00413-s001.zip › ijms-2085602-supplementary.pdf]

Bone Marrow-Derived Vasculogenic Mesenchymal Stem Cells Enhance In Vitro Angiogenic Sprouting of Human Umbilical Vein Endothelial Cells

Hyun Hee Jang [1], Youngsook Son [1], Gabee Park [1] and Ki-Sook Park [2,3,*]

[1] Graduate School of Biotechnology, Kyung Hee University, Yongin, Republic of Korea
[2] Department of Biomedical Science and Technology, Graduate School, Kyung Hee University, Seoul, Republic of Korea
[3] East-West Medical Research Institute, Kyung Hee University, Seoul, Republic of Korea
* Correspondence: kisookpark@khu.ac.kr; Tel.: +82-2-958-9368

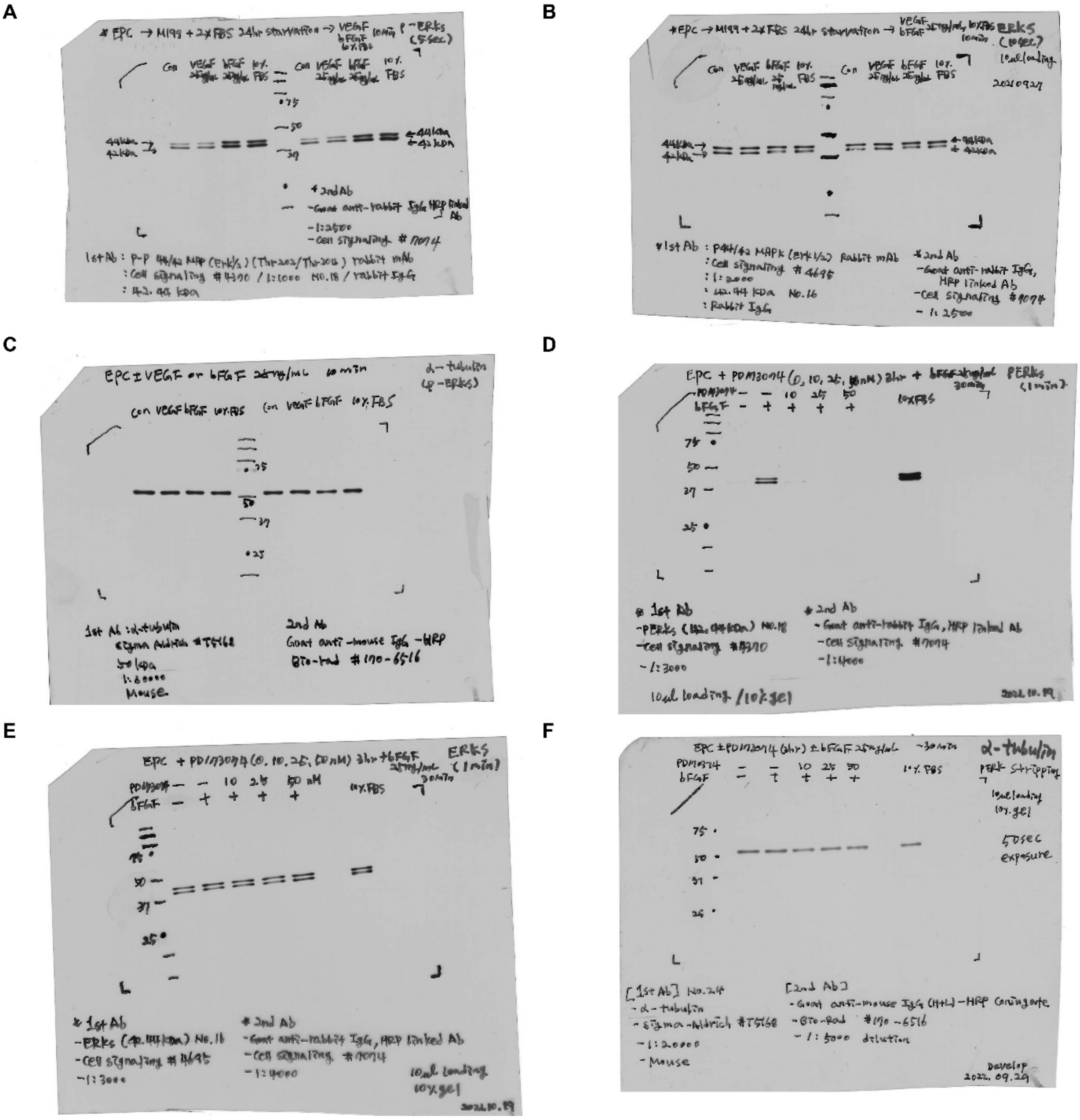

Supplementary Figure S1. Full unedited blots of Figure 1G and 1I. (A-C) Full unedited blots for Figures 1G; pERKs, ERKs, and α-tubulin. (D-F) Full unedited blots for Figure 1I; pERKs, ERKs, and α-tubulin.

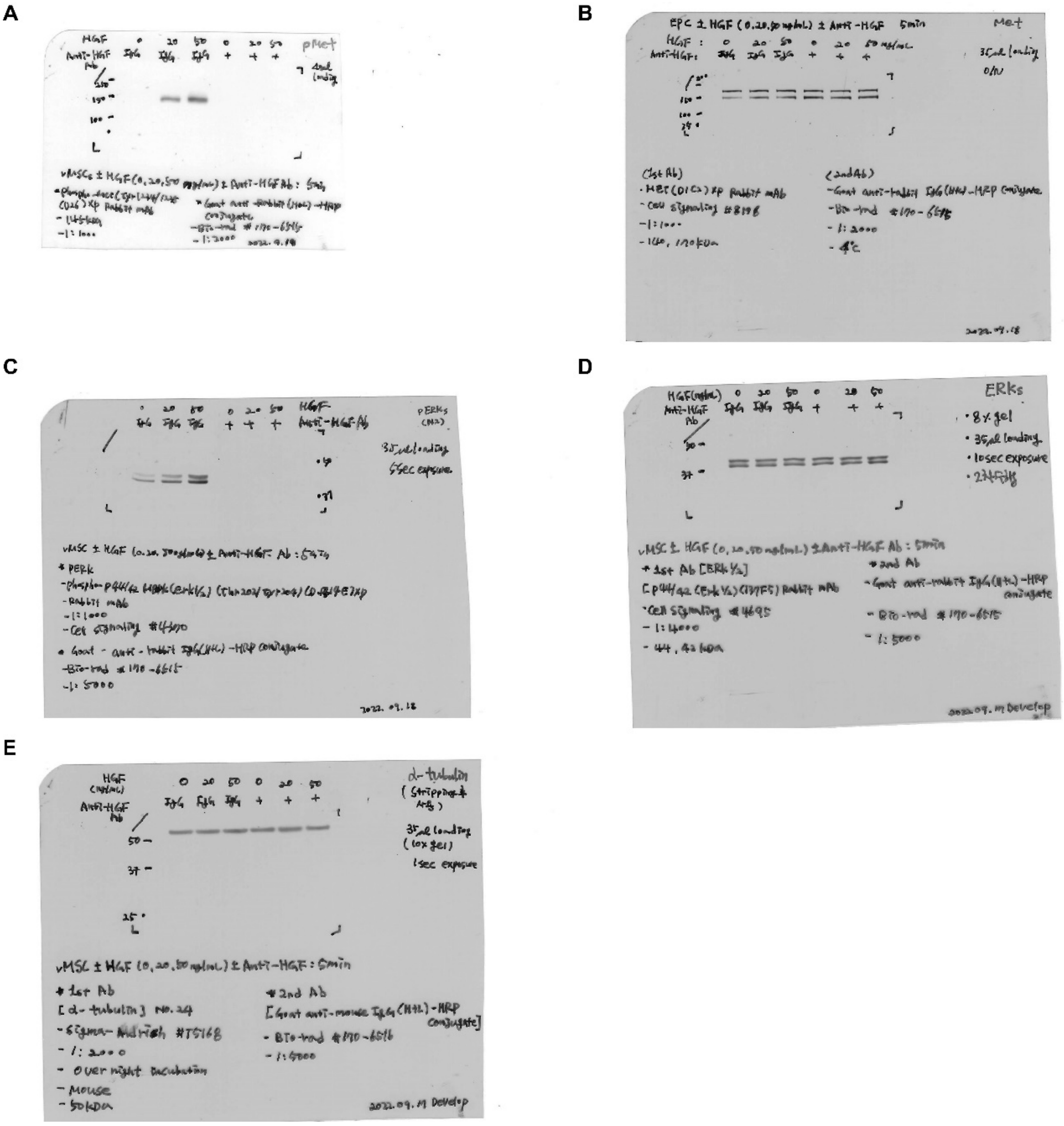

Supplementary Figure S2. Full unedited blots of Figure 2E. (A-E) phospho-c-Met, c-Met, pERKs, ERKs and α-tubulin.

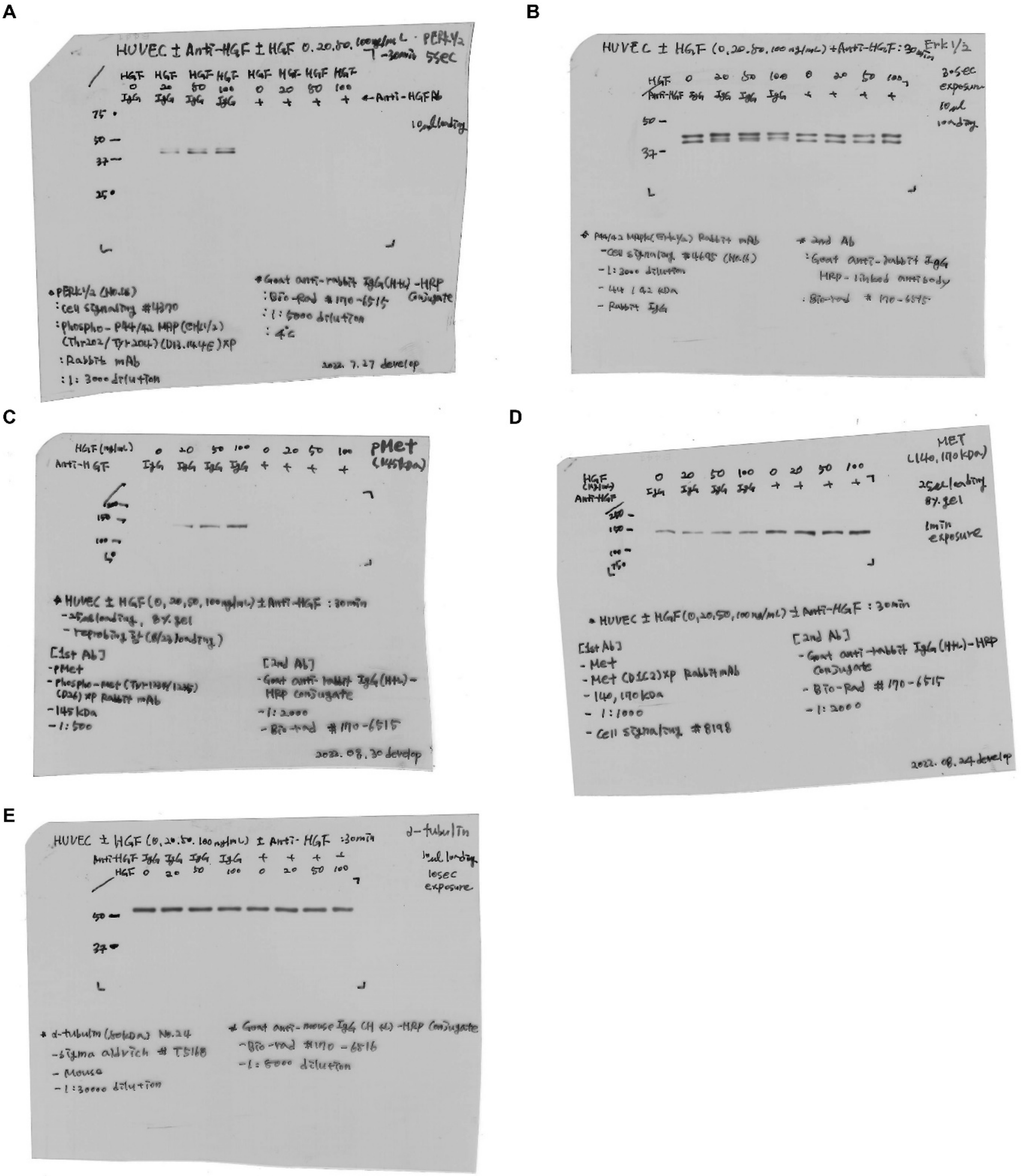

Supplementary Figure S3. Full unedited blots of Figure 4F. (A-E) phospho-c-Met, c-Met, pERKs, ERKs and α-tubulin.
